# ADJunctive Ulinastatin in Sepsis Treatment in China (ADJUST study): study protocol for a randomized controlled trial

**DOI:** 10.1186/s13063-018-2513-y

**Published:** 2018-02-21

**Authors:** Wei Jiang, Xiangyou Yu, Tongwen Sun, Yanfen Chai, Ping Chang, Zhongqing Chen, Jingye Pan, Zhiyong Peng, Ruilan Wang, Xiaozhi Wang, Yuan Xu, Li Yu, Qingshan Zheng, Bin Du, for the China Critical Care Clinical Trials Group (CCCCTG)

**Affiliations:** 1 Medical Intensive Care Unit, Peking Union Medical College Hospital, 1 Shuai Fu Yuan, Beijing, 100730 China; 2 Department of Critical Care Medicine, Xinjiang Medical University 1st Hospital, Urumqi, Xinjiang China; 3 Department of General Intensive Care Unit, the First Affiliated Hospital of Zhengzhou University, Zhengzhou, Henan China; 4 Department of Emergency, Tianjin Medical University General Hospital, Tianjin, China; 5 Department of Critical Care Medicine, Zhujiang Hospital of Southern Medical University, Guangzhou, Guangdong China; 6 Department of Critical Care Medicine, Nanfang Hospital of Southern Medical University, Guangzhou, Guangdong China; 7 Department of Critical Care Medicine, the First Affiliated Hospital of Wenzhou Medical University, Wenzhou, Zhejiang China; 8 Department of Critical Care Medicine, Zhongnan Hospital of Wuhan University, Wuhan, Hubei China; 9 Department of Critical Care Medicine, First People’s Hospital Affiliated to Shanghai Jiaotong University, Shanghai, China; 10 Department of Critical Care Medicine, Binzhou Medical University Hospital, Binzhou, Shandong China; 11 Department of Critical Care Medicine, Beijing Tsinghua Changgung Hospital Affiliated to Tsinghua University, Beijing, China; 12 Department of Critical Care Medicine, the Central Hospital of Wuhan Affiliated to Tongji Medical College Huazhong University of Science and Technology, Wuhan, Hubei China; 13 Centre for Drug Clinical Research, Shanghai University of Traditional Chinese Medicine, Shanghai, China

**Keywords:** Ulinastatin, Sepsis, Septic shock, Randomized controlled trial, Mortality, Safety

## Abstract

**Background:**

Sepsis is a major challenge in critical care and is associated with high mortality. Current management of sepsis and septic shock remains mainly supportive. Both basic and clinical research has shown that ulinastatin can improve the prognosis of sepsis. The aim of this trial is to evaluate the efficacy and safety profiles of ulinastatin compared with placebo.

**Methods/design:**

In this multi-center, double-blind, randomized placebo-controlled trial we are recruiting a total of 348 subjects meeting “The Third International Consensus Definitions for Sepsis and Septic Shock” (Sepsis-3). Subjects will be randomized (1:1) to receive ulinastatin 400,000 IU three times a day for 10 days or matching placebo and usual care simultaneously. The primary outcome is 28-day all-cause mortality. Adverse events and serious adverse events will be monitored closely.

**Discussion:**

ADJUST is a large, multi-center, double-blind, randomized, parallel-group, placebo-controlled trial of ulinastatin in mainland China and is well-designed on the basis of previous studies. The results of this trial may help to provide evidence-based recommendations for treatment of sepsis.

**Trial registration:**

ClinicalTrials.gov, ID: NCT02647554. First registered on 27 December 2015, and last verified in December of 2016.

Protocol version: 2.1, verified on 19 July 2016.

## Background

Sepsis is the leading cause of death among patients in non-coronary intensive care units (ICUs) [1]. Despite the advances in sepsis management during the past decade, the mortality rate still remains higher than 30%, and up to 60% when septic shock presents [2]. In high-income countries, the population incidence rate for sepsis was 437 cases per 100,000 person-years, corresponding to a global estimate of 31.5 million cases, with potentially 5.3 million deaths annually [3]. We recently reported the standardized sepsis incidence and mortality of 461 and 79 cases per 100,000 person-years, respectively, in China [4].

Sepsis exerts pleiotropic effects, overwhelming the host anti-inflammatory mechanisms, resulting in a generalized acute inflammatory response with life-threatening vascular, endothelial, and organ dysfunction [5, 6]. Until recently, most research on sepsis has focused on blocking the initial dysregulated, cytokine-mediated proinflammatory response [7]. Unfortunately, numerous large clinical trials involving immunomodulatory agents in sepsis have yielded discouraging results, despite promising preliminary data from animal and preclinical studies, careful design, clearly defined inclusion criteria and thorough statistical analysis, such as recombinant platelet-activation factor acetylhydrolase and recombinant bactericidal/permeability-increasing protein (rBPI_21_) [8–10].

Urinary trypsin inhibitor, also known as ulinastatin or bikunin, is an acid-resistant glycoprotein and Kunitz-type serine protease inhibitor composed of 143 amino acid residues that can be found in human blood and urine. Ulinastatin can also prevent inflammation and cytokine-dependent signaling pathways possibly via the suppression of the phosphorylation of extracellular signal-regulated kinase 1/2, c-Jun N-terminal kinase, as well as NF-κB or early growth response protein-1 signaling in response to a diverse array of stresses such as oxidative stress and inflammatory stress [11–13].

In animal models of sepsis, ulinastatin could reduce both systemic and regional inflammatory responses, suppress lymphocyte apoptosis, increase production of anti-inflammatory cytokines, such as interleukin (IL)-10 and IL-13, and improve survival rate [14–18]. Some pilot studies showed a trend towards decreasing mortality and duration of hospitalization with ulinastatin in severe sepsis [19, 20]. A recent meta-analysis reported that compared with a control group, ulinastatin treatment could significantly decrease the 28-day mortality rate (26.9% (152/565) vs 41.6% (228/547), RR 0.64; 95% CI 0.54–0.75; *P* < 0.01); however, two out of eight enrolled trials were of low quality and some of them were combined with thymosin [21]. This meta-analysis included a recently published prospective, double-blind, multi-center randomized controlled trial (RCT) conducted in seven hospitals in India. In this trial, Karnad and colleagues [22] demonstrated that 28-day all-cause mortality was significantly reduced in the ulinastatin group (7.3% vs 20.3%, *P* = 0.045). However, Karnad’s population was at relatively lower risk, with mean age below 40 years and a mean APACHE II score < 15 in both treated and placebo groups. The mortality in placebo group was only 20.3% (12/59), much lower than those in countries of similar socioeconomic status, such as 28.7% in China [23] and 55.7% in Brazil [24]. On the other hand, there were many more cases of multi-drug-resistant infection in the placebo group (12 vs 6, placebo and ulinastatin, respectively), which might cause more deaths in the placebo group. The modified intention-to-treat (ITT) population was another problem. The primary endpoint showed significant reduction of 28-day all-cause mortality in the modified ITT population. However, in the ITT population the reduction in mortality was from 20.6% (13/63) to 10.2% (6/59), which was short of statistical significance (*p* = 0.11). The modification came from 4 each discontinued intervention in both arms including one and two deaths from the placebo and ulinastatin groups, respectively, and the rationale for the withdrawal of patients after randomization for early mortality was unconvincing. Their inclusion renders the trial results statistically insignificant. All these have raised concerns over the internal and external validity of the study, which significantly hampers the generalization of the study results.

We therefore designed this study to evaluate the 28-day mortality of ulinastatin treatment compared with placebo in patients with sepsis in China.

## Methods/design

### Study design

The ADJUST study is designed as a prospective, multi-center, double-blind, randomized, parallel-group, placebo-controlled (ulinastatin vs placebo 1:1) superiority trial in 12 tertiary care hospitals. The participating sites in this study include medical/surgical, medical, surgical, or emergency ICUs (see Table 1 for details of participating centers).

**Table 1:** Research settings and names of each ethics committee

Research setting	Ethics committee name	Approval registration number
Peking Union Medical College Hospital	Medical Ethics Committee of Peking Union Medical College Hospital	HS-930
Xinjiang Medical University 1st Hospital	Medical Ethics Committee of Xinjiang Medical University 1st Hospital	D150827–02
Nanfang Hospital of Southern Medical University	Medical Ethics Committee of Nanfang Hospital of Southern Medical University	NFEC-201701-K3–01
Zhujiang Hospital of Southern Medical University	Medical Ethics Committee of Zhujiang Hospital of Southern Medical University	2016-ZZYXK-001
Tianjin Medical University General Hospital	Medical Ethics Committee of Tianjin Medical University General Hospital	IRB2017–008-01
Zhongnan Hospital of Wuhan University	Medical Ethics Committee of Zhongnan Hospital of Wuhan University	药伦[2016022]
First People’s Hospital Affiliated to Shanghai Jiaotong University	Medical Ethics Committee of First People’s Hospital Affiliated to Shanghai Jiaotong University	院伦审[2016]41号
the Central Hospital of Wuhan Affiliated to Tongji Medical College Huazhong University of Science and Technology	Medical Ethics Committee of the Central Hospital of Wuhan Affiliated to Tongji Medical College Huazhong University of Science and Technology	(2016)015号
the First Affiliated Hospital of Zhengzhou University	Medical Ethics Committee of the First Affiliated Hospital of Zhengzhou University	科研会审2016–49
Binzhou Medical University Hospital	Medical Ethics Committee of Binzhou Medical University Hospital	2017–005-01
the First Affiliated Hospital of Wenzhou Medical University	Medical Ethics Committee of the First Affiliated Hospital of Wenzhou Medical University	药伦审(2017)第012号
Beijing Tsinghua Changgung Hospital Affiliated to Tsinghua University	Medical Ethics Committee of Beijing Tsinghua Changgung Hospital Affiliated to Tsinghua University	17,081–0110

Patient enrollment is expected to last for up to 36 months. The end of the study is defined as the last follow-up of the last enrolled patient. The trial has been registered at ClinicalTrials.gov (NCT02647554).

### Recruitment

A well-trained study coordinator in each participating center will be responsible for screening all potentially eligible patients based on the eligibility criteria. After confirmation of the patient eligibility for the trial together with the treating physician, the study coordinator will then obtain written informed consent from the patient or authorized representatives.

### Inclusion criteria

Patients are eligible for the trial if they meet the criteria of “The Third International Consensus Definitions for Sepsis and Septic Shock” (Sepsis-3) and are between the age of 18 to 80 years [6]. The trial will include patients with confirmed or suspected infection and an acute change in total Sequential Organ Failure Assessment (SOFA) score ≥ 2 points [25]. Patients will be enrolled within 48 h of their fulfilling Sepsis-3 criteria.

### Exclusion criteria

Patients will be excluded if they meet any of the following criteria: (1) age below 18 or above 80 years; (2) pregnancy or lactation; (3) New York Heart Association Class IV chronic heart failure [26], or myocardial infarction within the previous 3 months; (4) uncontrolled blood loss; (5) cardiogenic shock; (6) advanced pulmonary fibrosis; (7) respiratory failure requiring non-invasive mechanical ventilation before enrollment; (8) severe, preexisting, parenchymal liver disease with clinically significant portal hypertension, Child-Pugh C stage cirrhosis [27], or acute liver failure [28]; (9) recipient of solid-organ or bone marrow transplant; (10) cardiopulmonary resuscitation within 72 h before enrollment; (11) invasive fungal infection [29, 30]; (12) active pulmonary tuberculosis [31]; (13) full-thickness thermal or chemical burn involving 30% or more of the body surface area; (14) evidence of clinically significant immunosuppression; (15) previous treatment with immunomodulatory agents, such as *Xuebijing*, thymosin, or intravenously administered immunoglobulin (IVIG) within 2 months before enrollment; (16) participation in an investigational clinical trial within 6 months before screening; (17) expected survival of less than 2 months or chronic vegetative state; (18) lack of commitment to full, aggressive life support; (19) history of hypersensitivity to ulinastatin. Clinically significant immunosuppression includes, but is not limited to, moderate or severe neutropenia (i.e., absolute neutrophil count < 1.0 × 10^9^/L), high-dose corticosteroids (corticosteroid equivalent of ≥ 20 mg prednisone every day for at least 2 weeks) prior to enrollment, immunomodulatory medications (e.g., cyclosporine, azathioprine, or OKT3), chemotherapy, or radiotherapy within 2 months before enrollment, known HIV seropositivity, non-remission stage of hematological/lymphoid malignancy, and any disease sufficiently advanced to suppress resistance to infection.

### Study drugs, drug distribution and storage

All study drugs and placebos are prepared by Guangdong Techpool Biopharmaceutical Co, Ltd. according to Good Manufacturing Practice guidelines. Blinded drug (lot 031507014) or placebo (lot P031506023) are supplied as lyophilized powders in single-dose vials containing ulinastatin 100,000 IU, or inactive ingredients (i.e., mannitol, sodium chloride, and phosphate buffer), respectively. Both study drug and placebo are shipped to each participating center in cartons containing 12 vials for a 1-day supply, and stored in a cool, well-ventilated place avoiding direct sunlight.

### Randomization

Following informed consent and confirmation of inclusion and exclusion criteria, participants will be randomly assigned in a 1:1 ratio to receiving either ulinastatin or placebo, with the random assignments stratified according to institution and presence of septic shock. The definition of septic shock is sepsis and hypotension requiring vasopressor support to maintain a mean blood pressure of 65 mmHg or greater as well as having a serum lactate level greater than 2 mmol/L after adequate fluid resuscitation. Dynamic randomization will be carried out using a Distribution Annotation Interactive Web Response System through a central, secured website: https://iwrs104.drugchina.org. Minimization as the most common covariate-adaptive randomization was adopted in our trial, which derived from the method of Pocock and Simon [32]. The first subject will be assigned according to the simple randomization, then the allocation that produces the least total imbalance is chosen with a higher probability (*p* = 0.8) for each subject after the first one. Among the previous study the probability of *p* = 0.8 has been shown to be the most efficient [33]. The reason that we used minimization in the design is that the balance of 12 centers cannot be guaranteed by the conventional randomization, and different hospitals will be considered as a covariate in the statistical analysis. The allocation is blinded to both subjects and investigators, as well as nurses and clinical research coordinators. During the treatment period, the investigator will log on to the above website every day and be given a study drug box number. With this number, the investigator could pick out the study drug box for the specific day from local storage.

The investigator will unblind allocation to the ulinastatin or placebo group should treatment of study-related adverse events (AEs) require it. The unblinding process will be well documented and reported to the principle investigator, the clinical research coordinator, and the administrative staff, when necessary. Unblinded subjects will be included in the primary, ITT analysis.

### Interventions

The study group and the control group will receive ulinastatin (400,000 IU every 8 h) and placebo, respectively, for 10 days, with the first dose administered within 8 h of study enrollment. In particular, the study drug is reconstituted with 10 ml of normal saline, which will be further diluted in 100 ml of normal saline. The study drug is administered over 1 to 2 h with the use of specially designed administration sets to ensure blinding. Matching placebo vials, with identical reconstitution and infusion instructions to the ulinastatin vials, will be administered on the same schedule. Apart from ulinastatin treatment, all enrolled patients will receive standard care for sepsis and septic shock as per local protocols, including, but not limited to, antimicrobial agents, intravenously administered fluids, enterally or parenterally administered nutrition, transfusion of blood and blood products, and life-sustaining therapy (e.g., vasopressors, mechanical ventilation, and renal replacement therapy). The treating physicians are encouraged to adhere to the updated Surviving Sepsis Campaign guidelines [34], although the final decision is at the discretion of the treating physicians.

Doses of ulinastatin showed significant variation in different clinical trials, from 300,000 IU [35] to 1,200,000 IU [36] daily. In a surveillance study of 17 ICUs of China Critical Care Clinical Trials Group (CCCCTG), 15 reported the use of ulinastatin as adjunctive treatment of sepsis, with daily dose range 200,000 IU to 2,000,000 IU (Bin Du, personal communication). The safety and tolerance of multi-dose (up to 1,200,000 IU qid) of ulinastatin have been evaluated in healthy volunteers without any serious adverse events (SAEs) [37]. In a rat model of sepsis, treatment with high-dose ulinastatin (200,000 IU/kg) significantly inhibited the production of TNF-α, P-selectin, and thrombin-antithrombin complex compared with a low dose (50,000 IU/kg) [38]. In a previous clinical study, a high dose of ulinastatin (300,000 IU twice a day) was associated with a significantly better clinical outcome and cytokine assays compared with middle- and low-dose ulinastatin (200,000 and 100,000 IU twice daily, respectively) [39]. The dose of ulinastatin in this trial was determined by consensus based on the above evidence.

### Concomitant interventions

Participants may receive stress-dose corticosteroids in the form of hydrocortisone at a daily dose of ≤ 300 mg for the treatment of refractory septic shock. In addition, the use of non-steroidal anti-inflammatory drugs is also acceptable for the purpose of defervescence according to the local protocol. Other immunomodulatory agents (e.g., *Xuebijing*, thymosin, and polyclonal IVIG) should not be used during the study period, except for the purpose of treating underlying diseases (e.g., IVIG for Guillain-Barré syndrome). Use of all above drugs, if any, should be documented in the electronic Case Report Form (eCRF). No other agents with anti-inflammatory effects are allowed during the study period.

### Outcome measurements

**Fig. 1:**
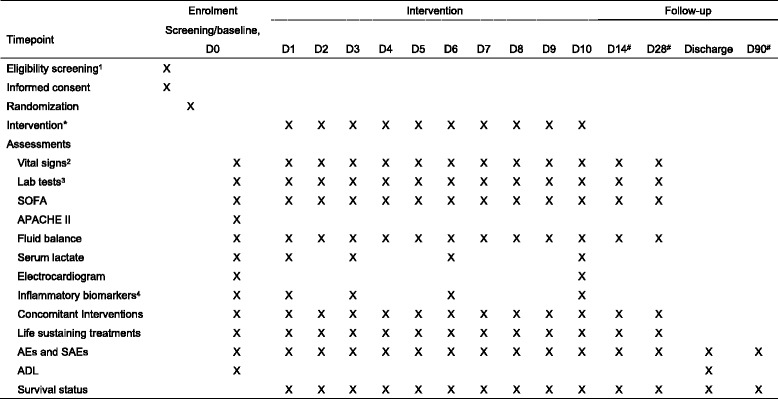
Trial schedule of the ADJUST study. Eligibility screening includes diagnosis, inclusion/exclusion criteria, urine pregnancy test for female, demographics, medical history and physical examination,as well as the presence of shock, ARDS, SIRS, Acute Circulatory Failure, time from diagnosis of sepsis, number of impaired organs. Vital signs include blood pressure, body temperature, heart rate, respiratory rate. Lab tests include complete blood count, serum alanine aminotransferase, aspartate aminotransferase and serum creatinine. Inflammatory biomarkers include hs-CRP, IL-6, TNF-α and IL-10, which are sent to central lab for test.*intervention means blinded ulinastatin 400000 IU 3 times a day or matching placebo and usual care.#The timeframes for D14, D28 and D90 allow deviations of 1, 3, 7 days, respectively.

Participants will be evaluated clinically and through laboratory testing according to Fig. 1.

The following data will be recorded: demographics, sepsis diagnosis, concurrent medical conditions and comorbidities, inclusion and exclusion criteria, severity of illness and organ dysfunction scores, vital signs and laboratory results, potential confounding co-interventions (life-sustaining therapies, and use of other immunomodulatory agents), and outcomes (vital status at ICU and hospital discharge, day 28 and day 90, ICU and hospital length of stay).

### Primary endpoint

The primary endpoint of this study is 28-day all-cause mortality.

### Secondary endpoints

The secondary endpoints of this trial include following:

1. Mortality analysis: including 90-day all-cause mortality, ICU mortality, hospital mortality, length of stay in ICU
2. Organ failure and supportive intervention requirements: including SOFA scores at baseline and days 1–10, 14, and 28; use of life-sustaining therapy (i.e., vasopressor, mechanical ventilation, renal replacement therapy); lactate levels at baseline and days 1, 3, 6, and 10; fluid balance during the first 10 days
3. Inflammatory biomarkers: including high-sensitivity C-reactive protein (hs-CRP), tumor necrosis factor (TNF)-α, IL-6, and IL-10 at baseline and days 1, 3, 6, and 10
4. Functioning: activities of daily living (ADL) level at baseline and hospital discharge [40]

We planned to collect data of organ dysfunction at baseline and various time points during the study. All the six domains of the SOFA score, including respiratory, coagulation, hepatic, cardiovascular, renal, and central nervous system, will be documented. Vasopressors, mechanical ventilation, and renal replacement therapy use will also be documented during the study as an assessment of organ dysfunction. New-onset organ dysfunction is defined as any increase of any domain in SOFA score.

Serum levels of inflammatory biomarkers (hs-CRP) and cytokines (IL-6, IL-10, and TNF-α) will be measured with a chemiluminescence assay and turbidimetric inhibition immunoassay, respectively, in a central laboratory (Kingmed Center for Clinical Laboratory). All the assay chipsets were manufactured by Siemens Healthcare Diagnostics, UK.

### Adverse events and serious adverse events

The safety profile of ulinastatin was an important endpoint of the current study, partially because of the relatively large dose we chose. According to the package inserts of ulinastatin for injection, adverse drug reactions are uncommon (0.1% to 1%), including neutropenia, eosinophilia, nausea, vomiting, diarrhea, increased alanine aminotransferase, increased aspartate aminotransferase, injection-site tenderness, flushing, pruritus, rashes, and anaphylaxis [41].

It is recognized that the patient with sepsis will experience a number of aberrations in laboratory values, signs, and symptoms due to the severity of the underlying disease and the impact of standard critical medicine therapies. These will not necessarily constitute an AE unless they are considered to be of related to study treatment or a concern in the investigator’s clinical judgement. In this study, reporting of AEs will be restricted to events that are considered to be related to study treatment (possibly, probably or definitely).

Serious adverse events are defined as any untoward medical occurrence that meets one of more of the following criteria:

- Results in death
- Is life-threatening
- Requires inpatient hospitalization or prolongation of existing hospitalization
- Results in persistent or significant disability/incapacity
- Is a congenital anomaly/birth defect

The treating physician will be responsible for determining the causal relationship of the SAE as either definitely, probably, possibly, possibly not, or definitely not study treatment-related, as well as unclassified.

All treatment-related AEs and SAEs should be recorded on eCRF. SAEs will be reported to the Institutional Ethics Committee within 24 h of study staff becoming aware of the events.

The participants are provided with commercial clinical research insurance by the manufacturer of the study product.

### Data management

Data management will be performed by trained staff at each participating center using the electronic data capture (EDC) system (https://edc702.drugchina.org). The quality of trial data management will be guaranteed by the reliability, access control, and traceability of the EDC system. Data collection will be restricted to those variables necessary to define baseline patient characteristics (demographics, sepsis diagnosis, concurrent medical conditions and comorbidities, inclusion and exclusion criteria, severity of illness and organ dysfunction scores, vital signs, and laboratory results), the delivery of the study drugs, potential confounding co-interventions (life-sustaining therapies, and use of other immunomodulatory agents), and outcomes (vital status at ICU and hospital discharge, day 28, and day 90, length of stay in ICU and hospital). Randomized participants will be followed up until either death or 90 days after randomization, whichever comes first. Follow-up will be attended by study staff by either direct contact with the patient or their next of kin. Participants who withdraw from the study for any reason will be followed up according to the study follow-up schedule and analyzed on the ITT principle.

### Data monitoring and auditing

An independent contract research organization (H&J CRO International, Inc.) will be responsible for data monitoring. The quality assurance division of the same contract research organization will audit the trial regularly. This trial will be also audited by each Research Ethics Committee and China Food and Drug Administration.

### Statistical analysis

#### Sample size estimation

We reported the hospital mortality rate of 33.5% in a 2-month prospective cohort study of 484 patients with severe sepsis/septic shock in China [23]. We estimate that 28-day all-cause mortality will be 33.5% in the control group. Treatment effect was difficult to predict. Several meta-analyses of RCTs had suggested that ulinastatin treatment was associated with an odds ratio (OR) of 0.57 (95% CI 0.34–0.95) in hospital mortality among patients with severe sepsis/septic shock, despite the fact that the treatment effect was statistically insignificant, and all included studies were of low quality and published in the Chinese language [20, 22, 35, 42–45]. Moreover, Karnad and colleagues reported that 28-day all-cause mortality was 7.3% with ulinastatin vs 20.3% with placebo, corresponding to a RRR of 0.64. Based on these results, we presume that a treatment effect with a RRR of 0.40 to 0.45 might be plausible estimates. We calculate that an enrollment of 348 participants will have a power of 80% to detect an absolute reduction of 14.5% (relative risk reduction of 42%) in the study group compared to 33.5% of the placebo group, allowing for a loss to follow-up or withdrawal of 20%. Considering the prevalence of severe sepsis/septic shock in our patients, and assuming the rate of patient recruitment of one to two cases per month per center (with 12 participating centers), this trial will be finished within 2 to 3 years.

### Data analysis

All analyses will be performed according to the ITT principle. A *P* value < 0.05 is considered statistically significant. All tests are two-sided with no adjustment for multiple comparisons. Continuous variables are reported as means and standard deviations or medians and interquartile ranges. Categorical variables are reported as proportions. Pearson’s chi-squared test and adjusted multivariable analysis will be applied for the primary outcome. We plan to perform subgroup analyses for the primary outcome for predefined variables: presence or absence of septic shock, time from onset of sepsis to study enrollment (≤ 24 h or 24 to 48 h), and concomitant use of corticosteroids (yes or no). All the other data including age, APACHE II score, acute respiratory distress syndrome (ARDS) [46], systemic inflammatory response syndrome [47], acute circulatory failure [48], and number of failed organs will be presented as descriptive results. Pearson’s chi-squared test will be used for comparison of mortality outcomes between groups. The *t* test will be used for comparison of SOFA scores, life-sustaining interventions, lactic acid levels, fluid balance, inflammatory biomarkers, and function indicators between groups. A paired *t* test will be used for comparison of baseline and changes during the intervention.

### Interim analysis

No interim analysis is planned for this trial.

## Discussion

Ulinastatin has been evaluated as a promising drug in the treatment of sepsis, due to the inhibition of various serine proteases, inflammatory mediators produced by neutrophils, and the production of TNF-α, IL-1, and IL-6 [49]. However, current evidence supporting the efficacy of ulinastatin in the treatment of sepsis, including RCTs, is subject to limitations such as a non-representative patient population, small sample size, and concomitant use of immunomodulatory agents (e.g., thymosin-α1). As a result, the effect of ulinastatin on the mortality of sepsis warrants validation by large, well-conducted RCTs in different settings [50].

At the time of preparation of the current study, ICU patients who met the new Sepsis-3 clinical criteria were reported to have a hospital mortality rate of 16.2 to 20.2% in high-income countries (HICs) [51, 52]. Such data were still lacking in middle- and low-income countries (MLICs). Furthermore, to what extent the above results might be generalized to MLICs remains unknown. Previous studies suggested that ICU patients with sepsis in MLICs might have a much higher mortality rate than those in HICs possibly due to inadequate resources and poor quality of care. Machado and colleagues reported a hospital mortality rate up to 55.7% among Brazilian patients with severe sepsis/septic shock [24]. In another Indian cohort, the 28-day mortality of sepsis was reported to be 62.8% [53]. As a result, using data from China [23], we estimate that 28-day all-cause mortality will be 33.5% in the control group.

The new definition of Sepsis-3 focused on organ function, so apart from the regular endpoints in clinical trials, such as mortality of various timeframes, we would like to employ more outcome measurements for organ function, together with mechanism and physical function. The four inflammatory biomarkers were chosen by the Trial Consensus Committee, and we expect that these data will add information on the mechanism of ulinastatin.

### Trial status

The first subject was enrolled on 5 December 2016. The trial is recruiting.

## Abbreviations

ADL: Activities of daily living
AE: Adverse event
APACHE: Acute Physiology and Chronic Health Evaluation
ARDS: Acute respiratory distress syndrome
CCCCTG: Chinese Critical Care Clinical Trials Group
eCRF: Electronic Case Report Form
EDC: Electronic data capture
HIC: High-income countries
ICU: Intensive care unit
IL: Interleukin
ITT: Intention-to-treat
IVIG: Intravenously administered immunoglobulins
MLIC: Middle- and low-income countries
RCT: Randomized controlled trial
SAE: Serious adverse event
SOFA: Sequential Organ Failure Assessment
TNF: Tumor necrosis factor
